# A lightweight, flow-based toolkit for parallel and distributed bioinformatics pipelines

**DOI:** 10.1186/1471-2105-12-61

**Published:** 2011-02-25

**Authors:** Marcin Cieślik, Cameron Mura

**Affiliations:** 1 Department of Chemistry, University of Virginia, Charlottesville, VA 22904-4319, USA; 2 Structural, Computational Biology & Biophysics, University of Virginia Health Sciences, Charlottesville, VA 22908, USA

## Abstract

**Background:**

Bioinformatic analyses typically proceed as chains of data-processing tasks. A pipeline, or 'workflow', is a well-defined protocol, with a specific structure defined by the topology of data-flow interdependencies, and a particular functionality arising from the data transformations applied at each step. In computer science, the dataflow programming (DFP) paradigm defines software systems constructed in this manner, as networks of message-passing components. Thus, bioinformatic workflows can be naturally mapped onto DFP concepts.

**Results:**

To enable the flexible creation and execution of bioinformatics dataflows, we have written a modular framework for parallel pipelines in Python ('PaPy'). A PaPy workflow is created from re-usable components connected by data-pipes into a directed acyclic graph, which together define nested higher-order map functions. The successive functional transformations of input data are evaluated on flexibly pooled compute resources, either local or remote. Input items are processed in batches of adjustable size, all flowing one to tune the trade-off between parallelism and lazy-evaluation (memory consumption). An add-on module ('NuBio') facilitates the creation of bioinformatics workflows by providing domain specific data-containers (*e.g*., for biomolecular sequences, alignments, structures) and functionality (*e.g*., to parse/write standard file formats).

**Conclusions:**

PaPy offers a modular framework for the creation and deployment of parallel and distributed data-processing workflows. Pipelines derive their functionality from user-written, data-coupled components, so PaPy also can be viewed as a lightweight toolkit for extensible, flow-based bioinformatics data-processing. The simplicity and flexibility of distributed PaPy pipelines may help users bridge the gap between traditional desktop/workstation and grid computing. PaPy is freely distributed as open-source Python code at http://muralab.org/PaPy, and includes extensive documentation and annotated usage examples.

## Background

Workflows are a natural model of how researchers process data [1], and will therefore only gain in relevance and importance as science continues becoming more data- and information-intensive. Unlike business workflows, which emphasize process modeling, automation and management, and are *control-flow *oriented [2,3], scientific pipelines emphasize *data-flow*, and fundamentally consist of chained transformations of collections of data items. This is particularly true in bioinformatics (see, *e.g*., [4] and references therein), spurring the recent development of workflow managment systems (WMS) to standardize, modularize, and execute *in silico *protocols. Such systems generally enable the construction, automation, deployment, exchange, re-use, and reproducibility of data-processing/analysis tasks [5]; catalogs of bioinformatically-capable WMS and web service (WS)-related systems can be found in relatively recent reviews [6,7].

The feature sets of existing WMS solutions vary in terms of monitoring, debugging, workflow validation, provenance capture, data management and scalability. While some WMS suites (*e.g*., BIOWMS [8], ERGATIS[9]) and pipelining solutions (*e.g*., CYRILLE2 [10]) are tailored to the bioinformatics domain, many serve as either general-purpose, domain-independent tools (*e.g*., KEPLER[3] and its underlying PTOLEMYII system [11], TAVERNA[12], KNIME[13]), frameworks for creating abstracted workflows suitable for enactment in grid environments (*e.g*., PEGASUS[14]), high-level "enactment portals" that require less programming effort by users (*e.g*., BIOWEP[15]), or flower-level software libraries (*e.g*., the Perl-based BIOPIPE[16]). Indeed, the recent proliferation of WMS technologies and implementations led Deelman *et al. *[5] to systematically study a taxonomy of features", particularly as regards the four stages of a typical workflow's lifecycle - creation, mapping to resources, execution, and provenance capture. The division into *task-based *versus *service-based *systems appears to be fundamental [5]. Systems of the first kind emphasize the orchestration and execution of a workflow, while the latter focus on service discovery and integration. With its emphasis on enabling facile creation of Python-based workflows for data processing (rather than, *e.g*., WS discovery or resource brokerage), PaPy is a *task-based *tool.

Traditional, non-WMS solutions for designing, editing, and deploying workflows are often idiosyncratic, and require some form of scripting to create input files for either a Make-like software build tool or a compute cluster task scheduler. Such approaches are, in some regards, simpler and more customizable, but they lack the aforementioned benefits of workflow systems; most importantly, manual approaches are brittle and in flexible (not easily sustainable, reconfigurable, or reusable), because the data-processing logic is hardwired into 'one-off ' scripts. At the other extreme, a common draw-back of integrated WMS suites is that, for transformations outside the standard repertoire of the particular WMS, a user may need to program custom tasks with numerous (and extraneous) adaptor functions ('shims' [17,18]) to finesse otherwise in-compatible data to the WMS-specific data-exchange format. This, then, limits the general capability of a WMS in utilizing ('wrapping') available codes to perform various, custom analyses. PaPy is a Python programming library that balances these two extremes, making it easy to create data-processing pipelines. It provides many of the benefits of a WMS (modular workflow composition, ability to distribute computations, monitoring execution), but preserves the simplicity of the Make-style approach and the flexibility of a general-purpose programming language. (PaPy-based workflows are written in Python.) The application programming interface (API) of PaPy reflects the underlying flow-based programming paradigm [19], and therefore avoids any impedance mismatch" [20] in expressing workflows. This enables PaPy to expose a compact, yet flexible and readily extensible, user interface.

Flow-based programming (FBP) and related approaches, such as dataflow programming languages [2], define software systems as networks of message-passing components. Discrete data items pass (as 'tokens') between components, as specified by a connection/wiring diagram; the runtime behavior (concurrency, deadlocks, *etc*.) of such systems may be analyzed via formal techniques such as Petri nets [21]. Most importantly for bioinformatics and related scientific domains, the individual pipeline components are coupled only by virtue of the pattern of data traversal across the graph and, therefore, the functions are highly modular, are insulated from one another, and are re-usable. The connections are defined independently of the processing components. Thus, flow-based programs can be considered as (possibly branched) data-processing assembly lines. Dataflow programming lends itself as a model for pipelining because the goal of modular data-processing protocols maps naturally onto the concept of components and connections. The input stream to a component consists of self-contained (atomic) data items; this, together with loose coupling between processing tasks, all flows for relatively easy parallelism and, consequently, feasible processing of large-scale datasets.

In PaPy, workflows are built from ordinary, user-definable Python functions with specific, well-defined signatures (call/return semantics). These functions define the operations of an individual PaPy processing node, and can be written in pure Python or may 'wrap' entirely non-Python binaries/executables. Thus, there are literally no arbitrary constrains on these functions or on a PaPy pipeline, in terms of functional complexity, utilized libraries or wrapped third-party programs. In this respect, PaPy is agnostic of specific application domains (astronomy, bioinformatics, cheminformatics, *etc*.). An auxiliary, independent module ('NuBio') is also included, to provide data-containers and functions for the most common tasks involving biological sequences and structures.

## Implementation

### Overview

PaPy has been implemented as a standard, cross-platform Python (CPython 2.6) package; the Additional File 1 (§3.1) provides further details on PaPy's platform independence, in terms of software implementation and installation. PaPy's dataflow execution model can be described, in the sense of Johnston *et al. *[2], as a *demand-driven *approach to processing of data streams. It uses the multiprocessing package [22] for local parallelism (*e.g*., multi-core or multi-CPU workstations), and a Python library for remote procedure calls (RPyC [23]) to distribute computations across networked computers. PaPy was written using the dataflow and object-oriented programming paradigms, the primary design goal being to enable the logical construction and deployment of workflows, optionally using existing tools and code-bases. The resulting architecture is based on well-established concepts from functional programming (such as higher-order 'map' functions) and workflow design (such as directed acyclic graphs), and naturally features parallelism, arbitrary topologies, robustness to exceptions, and execution monitoring. The exposed interface all flows one to define what the data-processing components do (workflow functionality), how they are connected (workflow structure) and where (upon what compute resources) to execute the workflow. These three aspects of PaPy's functionality are orthogonal, and therefore cleanly separated in the API. This construction promotes code re-use, clean workflow design, and alllows de-ployment in a variety of heterogenous computational environments.

### Modular design

The PaPy toolkit consists of three separate packages (Table 1) - PaPy, NuMap, NuBio - that provide Python modules (papy, numap, nubio) with non-overlapping functionality, and which can be utilized independently. The 'papy' module provides just four classes (Worker, Piper, Plumber, Dagger) to enable one to construct, launch, monitor and interact with workflows (Table 2 and Additional File 1 §3.2). To facilitate the construction of bioinformatics workflows with only minimal external dependencies, a 'nubio' module provides general data structures to store and manipulate biological data and entities (*e.g*., sequences, alignments, molecular structures), together with parsers and writers for common file formats. (This functionality is further described below.)

**Table 1:** Overview of the PaPy package

Package	Purpose
papy	Provides the core objects and methods for workflow construction and deployment, including the Worker, Piper, Dagger, and Plumber classes (see Table 2).

numap	Supplies an extension of Python's 'imap' facility, enabling parallel/distributed execution of tasks, locally or remotely (see Fig. 1B).

nubio	Provides data-structures and methods specific to bioinformatic data (molecular sequences, alignments, phylogenetic trees, 3 D structures)

PaPy is comprised of three packages, independently providing the set of functionalities described in this table.

**Table 2:** PaPy's core components (classes) and their roles

Component	Description & function
Piper, Worker	The core components (processing nodes) of a pipeline. User-defined functions (or external programs) are wrapped as Worker instances; a Piper wraps a Worker and, in conjunction with numap, further species the mode of evaluation (serial/parallel, local/remote, *etc*.); these key pipeline elements also provide exception-handling, logging, and produce/spawn/consume functionality.

Dagger	Defines the data-flow pipeline in the form of a DAG; allows one to add, remove, connect pipers, and validate topology. Coordinates the starting/stopping of NuMaps.

Plumber	High-level interface to run & monitor a pipeline: Provides methods to save/load pipeline code, alter and monitor state (initiate/run/pause/stop/*etc*.), and save results. (See Additional file 1 §3.2 for more information on the subtle differences between the Plumber and Dagger classes.)

NuMap	Implements a process/thread worker-pool. Allows pipers to evaluate multiple, nested map functions in parallel, using a mixture of threads or processes (locally) and, optionally, remote RPyC servers.

The 'numap' module supplies a parallel execution engine, using a flexible worker-pool [24] to evaluate multiple map functions in parallel. Used together with papy, these maps comprise some (or all) of the processing nodes of a pipeline. Like a standard Python 'imap', numap applies a function over the elements of a sequence or iterable object, and it does so lazily. Laziness can be adjusted via 'stride' and 'buffer' arguments (see below). Unlike imap, numap supports *multiple pairs *of functions and iterable *tasks*. The tasks are not queued, but rather are *interwoven *and share a pool of *worker *'processes' or 'threads', and a memory 'buffer'. Thus, numap's parallel (thread- or process-based, local or remote), buffered, multi-task functionality extends standard Python's built-in 'itertools.imap' and 'multiprocessing.Pool.imap' facilities.

### Workflow construction

A generic pipeline (Figure 1A) consists of *components *and *connections*. Components define data-processing tasks, while the topology of inter-connections coordinates the dataflow. Basic workflow patterns that have emerged [4] include those which are sequential (linear/unbranched or branched) or parallel (scatter/gather, MapReduce), those which incorporate decision logic (conditional branching), intricate loops or cyclic patterns, and so on. In PaPy, a workflow is a directed acyclic graph (DAG), with data-processing *nodes *and data-flow *edges*. The components are instances of a 'Piper' class, which are nodes of a 'Dagger' graph instance. A Dagger, in turn, is the DAG that literally defines the workflow connectivity. Processing nodes are constructed by wrapping user-provided functions into Worker instances. Together with a NuMap, the Worker is then wrapped to define a Piper (Figure 1C), instances of which are added as the nodes when composing a workflow graph. A function can be used within multiple nodes, and multiple functions can be chained or nested into higher-order functions within a single node (P(W(*f, g*,...)) in Figure 1A). Functions are easily shared between pipelines, and can be executed by remote processes because dependencies (import statements) are effectively 'attached' to the source-code specifying the function *via *Python decorators [25]. Execution engines are represented by NuMap objects (Figure 1B). Piper instances are optionally assigned to (possibly shared) NuMap instances that enable parallel execution. In terms of cloud computing, abstraction of compute resources in this manner should make PaPy workflows cloud-compatible (see Additional File 1 §3.3).

**Figure 1:**
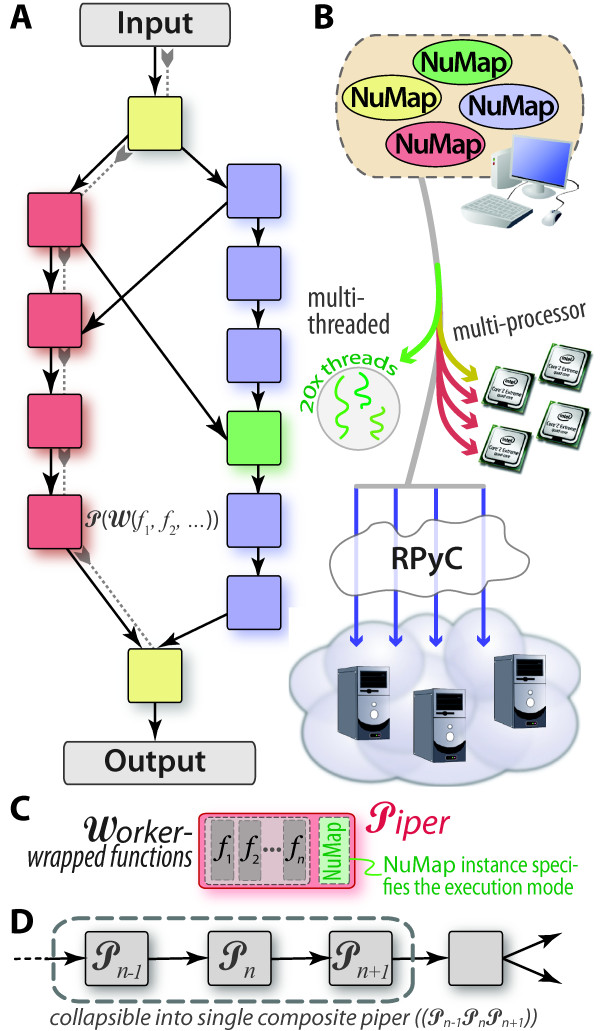
**A generic PaPy workflow**. Any generic workflow that is expressible as a directed graph can be implemented as a PaPy pipeline (A). As indicated by the pipes linking separate processing streams in (A), workflow construction in PaPy is flexible, not restrictive. Because of the methods that PaPy's NuMap objects can use to parallelize or distribute calculations (text, Table 3), a workflow can utilize a variety of available computational resources, such as threads, multi-processor architectures, and remote resources (B). PaPy's Dagger objects, representing the entire pipeline, are comprised of Piper nodes (colored squares) inter-connected via pipes (black arrows); 'pipes' can, equivalently, be considered as edges that represent data-flow dependencies (gray arrows 'pulling' data through the left branch of (A)). Colors are used to match sample Pipers (A) with their NuMap instances (B), and the conceptual relationship between Piper, Worker, and NuMap concepts is shown in (C). Parallelism is achieved by pulling data through the pipeline in adjustable batches, and overall performance may be improved by collapsing unbranched linear segments into a single node (D).

### Bioinformatics workflows

Because the architecture of PaPy is generalized, it is more of a software library than a single, domain-specific program, and it is therefore able to drive arbitrary workflows (bioinformatic or not). To enable rapid, consistent development, and facile deployment, of bioinformatics workflows, a lightweight package ('NuBio') is provided. NuBio consists of data structures to store and manipulate biological entities such as molecular sequences, alignments, 3 D structures, and trees. The data containers are based on a hierarchical, multi-dimensional array concept. Raw data are stored in at arrays, but the operational (context-dependent) meaning of a data-item is defined at usage, in a manner akin to NumPy's view casting" approach to subclassing *n*-dimensional arrays (see [26] and Example 2 below). For example, the string object 'ATGGCG' can act as a 'NtSeq' (sequence of six nucleotides) or as a 'CodonSeq' (sequence of two codons) in NuBio. This alllows one to customize the behaviour of objects traversing the workflow and the storage of metadata at multiple hierarchical levels. Functions to read and write common file formats are also bundled in PaPy (PDB for structural data, FASTA for sequences, Stockholm for sequence alignments, *etc*.).

### Parallelism

Parallel data-processing is an important aspect of workflows that either (*i*) deal with large datasets, (*ii*) involve CPU-intensive methods, or *(iii*) perform iterated, loosely-coupled tasks, such as in "parameter sweeps" or replicated simulations. Examples in computational biology include processing of raw, 'omics'-scale volumes of data (*e.g*. [27]), analysis/post-processing of large-scale datasets (*e.g*. molecular dynamics simulations in [28]), and computational approaches that themselves generate large volumes of data (*e.g*. repetitive methods such as replica-exchange MD simulations [29,30]). PaPy enables parallelism at the processing node and data-item levels. The former (node-level) corresponds to processing independent data items concurrently, and the latter (item level) to running parallel, independent jobs for a single data item.

**Figure 2:**
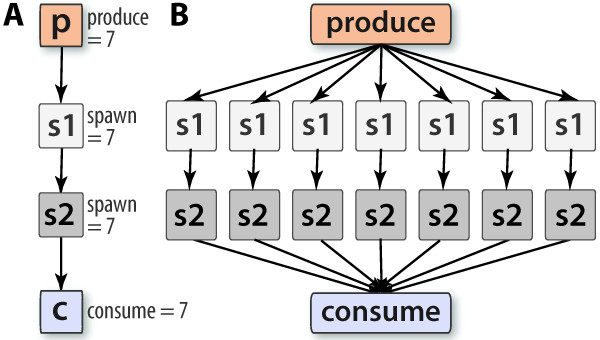
**The produce/spawn/consume idiom**. This workflow design pattern, used to process a single input node in parallel, arises in many contexts, such as in replica exchange simulations (see text). In this and remaining workflow diagrams (Figs. 3, 4), the sequence of Piper nodes is shown on the left (A), while the discrete data transformations that will implicitly occur (at the data-item level) are schematized on the right (B).

PaPy's parallelism is achieved using the worker-pool [24] design pattern, which is essentially an abstraction of the lower-level producer/consumer paradigm (see, *e.g*., [31,32]). Originally devised to address issues such as concurrency and synchronization in multi-programming, the produce/spawn/-consume idiom is useful at higher levels (such as dataflow pipelines), involving generation/processing of streams of data items (Figure 2). As schematized in Figure 1B, a NuMap instance uses a collection of local or remote computational resources (*i.e*., it abstracts a worker-pool) to evaluate, in parallel, one or several map functions. (A Piper instance becomes parallel if associated with a NuMap instance specifying parallel evaluation, or Python's itertools.i map.) Multiple pipers within a workflow can share a single worker-pool, and multiple worker-pools can be used within a workflow. This, together with the possibility of mixing serial and parallel processing nodes, allows for performance tuning and load-balancing. To benefit from parallel execution, the data-processing function should have a high granularity *i.e*., the amount of time spent per calculation is large compared to periods of communication. Note that this general approach bears similarity to the MapReduce model of distributed computing [33], and could be suitable for replicated, loosely-coupled tasks, such as in Monte Carlo sampling, replica-exchange MD simulations, or genome-wide motif searches (*e.g*. [34]).

### Dataflow

**Figure 3:**
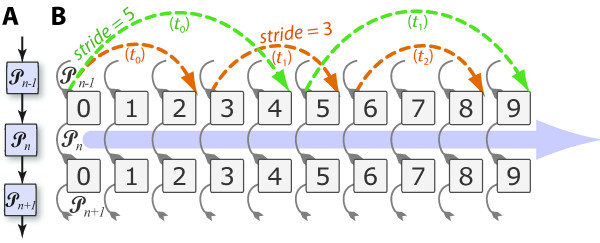
**The 'stride' as a control parameter**. PaPy's adjustable *stride *modulates the trade-off between high memory consumption and parallelism (high stride) versus less aggressive paralellism and lower memory consumption (lower stride). This diagram un-winds PaPy's parallelism to show the interplay between the *stride *and item-level processing as pipeline execution proceeds (main blue arrow directed rightward). The relevent pipers are shown to the left (A), and traversal of the workflow graph by data-batches is shown in (B). Execution progress is also indicated by broken arrows progressing to the right, each arc representing equal incremenets of time (*t*_0_, *t*_1_,.... assuming a uniform processing time per data-item) for strides of 3 (orange) or 5 (green).

The flow of data through a pipeline is intimately linked to the issue of parallelism. In PaPy, data traverse a pipeline in batches of a certain size, as defined by an adjustable '*stride' *parameter (Figure 3). The *stride *is the number of data items processed in parallel by a node in the workflow. The larger the stride the higher the scalability, as this results in fewer idle processes and greater speed-ups. However, memory requirements increase with batch size, as potentially more temporary results will have to be held in memory. Thus, the adjustable memory/speedup trade-off allows PaPy to deal with datasets too large to fit into resident memory and to cope with highly variable processing times for individual input items. Note that the order in which data items are submitted to the pool for evaluation is not the same as the order in which results become available; because of synchronization of processing nodes, this may cause a pipeline to incur idle CPU cycles. PaPy circumvents this potential inefficiency by (optionally) relaxing any requirement of ordered dataflow within a pipeline, as further described in the software documentation.

### Data-handling and serialization issues

Pipers must communicate the results computed by their wrapped functions. In PaPy's execution model, synchronization and message passing within a workflow are achieved by means of queues and locked pipes in the form of serialized Python objects. (*Serialization *refers to a robust, built-in means of storing a native Python object as a byte-string, thereby achieving object persistence.) Unlike heavyweight WMS suites such as KNIME (see the Additional File 1 §4), PaPy does not enforce a specific rigorous data exchange scheme or file format. This intentional design decision is based on the *type system *[35] of the Python programming language, whereby the valid semantics of an object are determined by its dynamic, user-modifiable properties and methods ("duck typing"). Such potentially polymorphic data structures cannot be described by, *e.g*., XML schema [36], but serialization offers a method of losslessly preserving this flexible nature of Python objects. In PaPy, component interoperability is achieved by adhering to duck-typing programming patterns. By default, no intermediate pipeline results are stored. This behavior can be easily changed by explicitly adding Piper nodes for data serialization (*e.g*. JSON) and archiving (*e.g*. files) anywhere within a workflow.

### Inter-process communication (IPC)

**Figure 4:**
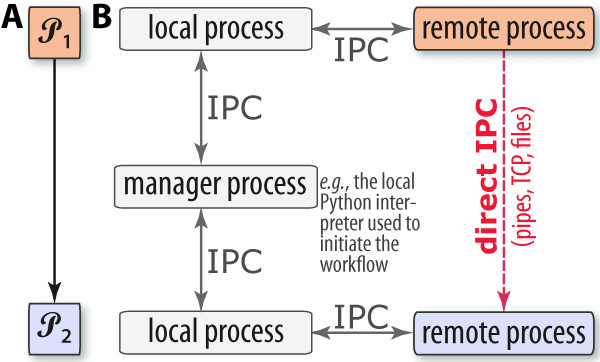
**Inter-process communication in PaPy**. The possible means of IPC between two linked pipers ($P_{1}$, $P_{2}$) in a PaPy graph are indicated (A), and the dashed allow (B) denotes the possibility of direct IPC via sockets, pipes, shared memory, *etc*. (Table 3). Communication between local and remote processes utilizes RPyC, as described in the text.

IPC may occur between a single local manager process (Figure 4), local pool processes/threads and, potentially, any remote processes (if operating in distributed mode, across networked machines). Data serialization and transmission is an important aspect, and often bottleneck, in parallel computing [37], because of the involved computational cost and utilized bandwidth. PaPy provides functionality for direct connections between processing nodes in order to mostly bypass the manager process (Figure 4). In essence, the mechanism is that the source component makes data available (*e.g*. by storing it as a file or opening a network socket) and communicates only the information needed to locate and access it by the destination component. PaPy provides a few mechanisms of direct IPC (files, Unix pipes, network sockets) as described in Table 3; an earlier implementation of PaPy, utilizing the posix_ipc shared memory library for direct IPC, was found to be no faster than Unix pipes. It is also possible to avoid IPC altogether, by grouping data-processing functions: A PaPy processing node is guaranteed to evaluate a single data item within the same process, meaning that no IPC occurs between functions within a single Piper instance. Thus, any linear, non-branching segment of a workflow can be easily collapsed into a single Piper node, as illustrated in Figure 1D. Default automation of this locality-enforcing behavior (*i.e*., automatically collapsing consecutive nodes in a linear segment of a pipeline) may be implemented in future versions of PaPy.

**Table 3:** PaPy's interprocess communication (IPC) methods

Method	OS	Remarks
socket	all	Communication, *via *TCP sockets, between hosts connected within a computer network

pipes	Unix like	Communication between processes on a single host

files	all	The file storage location must be accessible by all processes (*e.g*., over an NFS or Samba share).

PaPy provides the following direct IPC methods (see also Fig. 4), valid on operating systems as indicated.

### Monitoring

Interactive, real-time viewing of execution progress is valuable for parallel programs in general (*e.g*. for purposes of debugging), and it is particularly useful in workflow execution and editing to be able to log component invocations during the workflow lifecycle [5]. The information should be detailed enough to allow troubleshooting of errors and performance issues or auditing, and is a key aspect of the general issue of data provenance (data and metadata recording, management, workflow reproducibility). The process of capturing information about the operation of an application is often called 'logging'. For this purpose, PaPy utilizes the Python standard library's 'logging' facility, and automatically records logging statements emitted at various (user-specifiable) levels of detail or severity - *e.g*., DEBUG, INFO, WARNING, ERROR can be logged by the papy and numap modules. Python supplies rich exception-handling capabilities, and user-written functions need only raise meaningful exceptions on errors in order to be properly monitored.

### Robustness

Sooner or later in the life-cycle of a workflow, an error or exception will occur. This will most likely happen within a Worker-wrapped function as a result of bogus or unforseen input data, timeouts, or bugs triggered in external libraries. PaPy is resilient to such errors, insofar as exceptions raised within functions are caught, recorded and wrapped into 'placeholders' that traverse the workflow down-stream without disrupting its execution. The execution log will contain information about the error and the data involved.

## Results & Discussion

While a thorough description of PaPy's usage, from novice to intermediate to advanced levels, lies beyond the scope of this article, the following sections *(i) *illustrate some of the basic features of PaPy and its accompanying NuBio package (Examples 1, 2, 3), (*ii*) provide heavily-annotated, generic pipeline templates (see also Additional File 1), *(iii) *outline a more intricate PaPy workflow (simulation-based loop refinement, the details of which are in the Additional File 1), and (*iv*) briefly consider issues of computational efficiency.

### Example 1: PaPy's Workers and Pipers

The basic functionality of a node (Piper) in a PaPy pipeline is literally defined by the node's Worker object (Table 2 and the *'W *' in Figure 1A). Instances of the core Worker class are constructed by wrapping functions (user-created or external), and this can be done in a highly general and flexible manner: A Worker instance can be constructed *de novo *(as a single, user-defined Python function), from multiple pre-defined functions (as a tuple of functions and positional or keyworded arguments), from another Worker instance, or as a composition of multiple Worker instances. To demonstrate these concepts, consider the following block of code:

from papy import Worker

from math import radians, degrees, pi

def papy_radians(input): return radians(input[0])

def papy_degrees(input): return degrees(input[0])

worker_instance1 = Worker(papy_radians)

worker_instance1([90.]) # returns 1.57 (i.e., pi/2)

worker_instance2 = Worker(papy_degrees)

worker_instance2([pi]) # returns 180.0

# Note double parentheses (tuple!) in the following:

worker_instance_f1f2 = Worker((papy_radians, papy_degrees)) worker_instance_f1f2([90.]) # returns 90. (rad/deg invert!)

# Another way, compose from Worker instances:

worker_instance_w1w2 = *Worker*((worker_instance1,\worker_instance2))

# Yields same result as worker_instance_f1f2([90.]): worker_instance_w1w2([90.])

In summary, Worker objects fulfill several key roles in a pipeline: They (*i*) standardize the input/output of nodes (pipers); (*ii*) allow one to re-use and re-combine functions into custom nodes; (*iii) *provide a pipeline with graceful fault-tolerance, as they catch and wrap exceptions raised within their functions; and (*iv*) wrap functions in order to enable them to be evaluated on remote hosts.

The following block of Python illustrates the next 'higher' level in PaPy's operation - Encapsulating Worker-wrapped functions into Piper instances. In addition to what is done (Workers), the Piper level wraps NuMap objects to define the mode of execution (serial/parallel, processes/threads, local/remote, ordered/unordered output, *etc*.); therefore, a Piper can be considered as the minimal logical processing unit in a pipeline (squares in Figure 1, 2A, 3A, 4A).

from papy import Worker, Piper from numap import NuMap

from math import sqrt

# Square-root worker:

def papy_sqrt(input): return sqrt(input[0])

sqrt_worker = Worker(papy_sqrt)

my_local_numap = NuMap() # Simple (default) NuMap instance

# Fancier NuMap worker-pool:

# my_local_numap = NuMap(worker_type ="thread",\

# worker_num = 4, stride = 4)

my_piper_instance = Piper(worker = sqrt_worker, \ parallel = my_local_numap)

my_piper_instance([1,2,3]).start() list(my_piper_instance)

# returns [1.0, 1.414..., 1.732...]

# following will *not *work, as piper hasn't been stopped:

my_piper_instance.disconnect()

# ...but nflow the call to disconnect *will *work:

my_piper_instance.stop() my_piper_instance.disconnect()

The middle portion (lines 7-12) of the above block of code illustrates two examples of NuMap construction, which, in turn, defines the mode of execution of a PaPy workflow - Either a default NuMap (line 7), or one that specifies multi-threaded parallel execution using four workers (lines 9-12).

### Example 2: Basic sequence objects in NuBio

As outlined in the earlier *Bioinformatics workflows *section, the NuBio package was written to extend PaPy's feature set by including basic support for handling biomolecular data, in as flexible and generalized a manner as possible. To this end, NuBio represents all biomolecular data as hierarchical, multidimensional entities, and uses standard programming concepts (such as 'slices') to access and manipulate these entities. For instance, in this frame-work, a single nucleotide is a scalar object comprised of potentially *n*-dimensional entities (*i.e.*, a character), a DNA sequence or other nucleotide string is a vector of rank-1 objects (nucleotides), a multiple sequence alignment of *n *sequences is analogous to a rank-3 tensor (an (*n*-dim) array of (1-dim) strings, each composed of characters), and so on. The following blocks of code tangibly illustrate these concepts (output is denoted by '- > '):

from nubio import NtSeq, CodonSeq

from string import upper, lower

# A sequence of eight codons:

my_codons_1 = CodonSeq('GUUAUUAGGGGUAUCAAUAUAGCU')

# ...and the third one in it, using the 'get_child' method:

my_codons_1_3 = my_codons_1.get_child(2)

# ...and its raw (internal) representation as a byte string

# (ASCII char codes):

print my_codons_1_3

-> Codon('b', [65, 71, 71])

# Use the 'tobytes' method to dump as a char string: print my_codons_1_3.tobytes()

-> AGG

# 'get_items' returns the codon as a Python tuple: print my_codons_1.get_item(2)

-> ('A', 'G', 'G')

# The string 'UGUGCUAUGA' isn't a multiple of 3 (rejected

# as codon object), but is a valid NT sequence object:

my_nts_1 = NtSeq('UGUGCUAUGA')

# To make its (DNA) complement:

my_nts_1_comp = my_nts_http://1.complement() print my_nts_1_complement ()

-> ACACGATACT

# Sample application of a string method, rendering the

# original sequence lowercase (in-place modification):

my_nts_1.str(method="lower")

print my_nts_1.tobytes() -> ugugcuauga

# Use NuBio's hierarchical representations and data conta-

# iners to perform simple sequence(/string) manipulation:

# grab nucleotides 3-7 (inclusive) from the above NT string:

my_nts_1_3to7 = my_nts_1.get_chunk((slice(2, 7), slice(0,1))) print my_nts_1_3to7.tobytes()

-> ugcua

# Get all but the first and last (-1) NTs from the above NT

# string:

my_nts_1_NoEnds = my_nts_1.get_chunk((slice(1, -1), \ slice(0,1)))

print my_nts_1_NoEnds.tobytes()-> gugcuaug

# Get codons 2 and 3 (as a flat string) from the codon string:

my_codons_1_2to3 = my_codons_1.get_chunk((slice(1,3,1), \

slice(0,3,1)))

print my_codons_1_2to3.tobytes() -> AUUAGG

# Grab just the 3rd (wobble) position NT from each codon:

my_codons_1_wobble = my_codons_1.get_chunk((slice(0,10,1), n

slice(2,10,1)))

print my_codons_1_wobble.tobytes() -> UUGUCUAU

For general convenience and utility, NuBio's data structures can access built-in dictionaries provided by this package (*e.g*., the genetic code). In the following example, a sequence of codons is translated:

# Simple: Methionine codon, followed by the opal stop codon:

nt_start_stop = NtSeq("ATGTGA")

# Instantiate a (translate-able) CodonSeq object from this:

codon_start_stop = CodonSeq(nt_start_stop.data)

# ...and translate it:

print(codon_start_stop.translate()) ->

-> AaSeq(M*)

print(codon_start_stop.translate(strict = True))

-> AaSeq(M)

The follflowing block illustrates manipulations with protein sequences:

from nubio import AaSeq, AaAln

# Define two protein sequences. Associate some metadata (pI,

# MW, whatever) with the second one, as key/value pairs:

seq1 = AaSeq('MSTAP')

seq2 = AaSeq('M-TAP', meta='my_key':'my_data')

# Create an 'alignment' object, and print its sequences:

aln = AaAln((seq1, seq2))

for seq in aln: print seq

-> AaSeq(MSTAP)

-> AaSeq(M-TAP)

# Print the last 'seq' ("M-TAP"), sans gapped residues

# (i.e., restrict to just the amino acid ALPHABET):

print seq.keep(seq.meta['ALPHABET'])

-> AaSeq(MTAP)

# Retrieve metadata associated with 'my_key': aln[1].meta['my_key']

-> 'my_data'

### Example 3: Produce/spawn/consume parallelism

Loosely-coupled data can be parallelized at the data item-level *via *the produce/consume/spawn idiom (Figure 2). To illustrate how readily this workflow pattern can be implemented in PaPy, the source code includes a generic example in doc/examples/hello_produce_spawn_consume.py. The 'hello_*' files in the doc/examples/ directory provide numerous other samples too, including creation of parallel pipers, local grids as the target execution environment, and a highly generic workflow template.

### Generic pipeline templates

To assist one in getting started with bioinformatic pipelines, PaPy also includes a generic pipeline template (Additional File 1 §1.1; 'doc/workflows/pipeline.py') and a sample workflow that illustrates papy/nubio integration (Additional File 1 §1.2; 'doc/examples/hello_workflow.py'). The prototype pipeline includes commonly encountered workflow features, such as the branch/merge topology. Most importantly, the example code is annotated with descriptive comments, and is written in a highly modular manner (consisting of six discrete stages, as described in Additional File 1). The latter feature contributes to clean workflow design, aiming to decouple those types of tasks which are logically independent of one another *(e.g*, definitions of worker functions, workflow topology, and compute resources need not be linked).

### Advanced example: An intricate PaPy workflow

**Figure 5:**
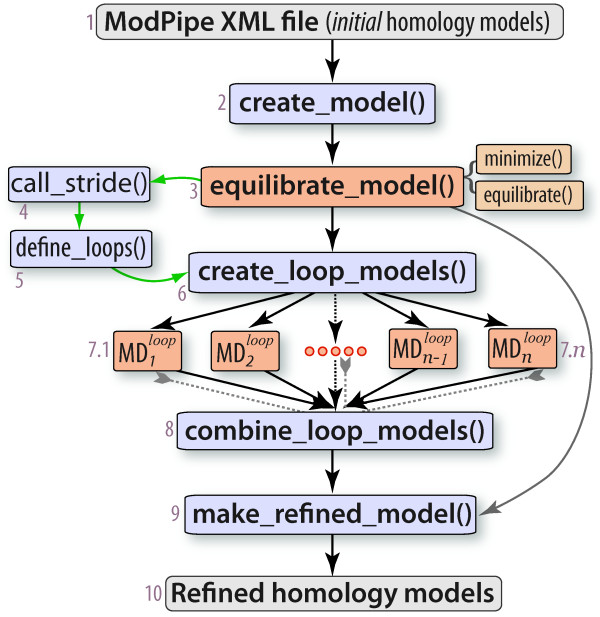
**MD-based loop refinement**. This pipeline illustrates a series of steps to perform MD simulation-based refinement of homology model loops, using the workflow paradigm. Piper nodes are numbered in this figure (for ease of reference), and can be classified into *(i*) those that handle input/output (grey; 1, 10); (*ii*) those that execute calculations serially (light blue; 2, 4, 5, 6, 8, 9); and (*iii) *more compute-intensive nodes, which utilize a parallel NuMap (orange; 3, 7). A detailed description of this use-case is available in the *Additional File *(§2).

In protein homology modelling, potentially flexible loop regions that link more rigid secondary structural elements are often difficult to model accurately (*e.g*. [38]). A possible strategy to improve the predicted 3 D structures of loops involves better sampling the accessible conformational states of loop backbones, often using simulation-based approaches (*e.g*. [39]). Though a complete, PaPy-based implementation of loop refinement is beyond the scientific scope of this work, we include a use-case inspired by this problem for two primary reasons: (1) The workflow solution demonstrates how to integrate third-party software packages into PaPy (*e.g*., Stride [40] to compute loop boundaries as regions between secondary structural elements, MMTK [41] for energy calculations and simulations); (2) Loop-refinement illustrates how an intricate structural bioinformatics workflow can be expressed as a PaPy pipeline. This advanced workflow demonstrates constructs such as nested functions, forked pipelines, the produce/s-pawn/consume idiom, iterative loops, and conditional logic. The workflow is schematized in Figure 5 and a complete description of this case study, including source code, can be found in Additional File 1 (§2 and Fig. S1, showing parallelization over loops and bounding spheres).

### Computational efficiency

Achieving speed-ups of workflow execution is non-trivial, as process-based parallelism involves *(i) *computational overhead from serialization; (*ii*) data transmission over potentially low-bandwidth/high-latency communication channels; (*iii) *process synchronization, and the associated waiting periods; and (*iv*) a potential bottelneck from the sole manager process (Figure 4). PaPy allows one to address these issues. Performance optimization is an activity that is mostly independent of workflow construction, and may include collapsing multiple processing nodes that preserves locality and increase granularity (Figure 1), employing direct IPC (Figure 4 Table 3), adjustments of speedup/memory trade-off parameter (Figure 3), allowing for unordered flow of data and, finally, balanced distribution of computational resources among segments of the pipeline. The PaPy documentation further addresses these intricacies, and suggests possible optimization solutions for common usage scenarios.

### Further information

In addition to full descriptions of the generic PaPy pipeline template and the sample loop-refinement workflow (Additional File 1), further information is available. In particular, the documentation distributed with the source-code provides extensive descriptions of both conceptual and practical aspects of workflow design and execution. Along with overviews and introductory descriptions, this thorough (≈50-page) manual includes *(i) *complete, step-by-step installation instructions for the Unix/Linux platform; (*ii*) a *Quick Introduction *describing PaPy's basic design, object-oriented architecture, and core components (classes), in addition to hands-on illustrations of most concepts *via *code snippets; *(iii*) an extensive presentation of parallelism-related concepts, such as maps, iterated maps, NuMap, and so on; (*iv*) a glossary of PaPy-related terms; and (*v*) because PaPy is more of a library than a program, a complete description of its application programming interface (API).

Although a thorough analysis of PaPy's relationship to existing workflow-related software solutions lies beyond the scope of this report, Additional File 1 (§4) also includes a comparative overview of PaPy, in terms of its similarities and differences to an example of a higher-level/heavyweight WMS suite (KNIME).

## Conclusions

PaPy is a Python-based library for the creation and execution of cross-platform scientific workflows. Augmented with a 'NuMap' parallel execution engine and a 'NuBio' package for generalized biomolecular data structures, PaPy also provides a lightweight tool for data-processing pipelines that are specific to bioinformatics. PaPy's programming interface reflects its underlying dataflow and object-oriented programming paradigms, and it enables parallel execution through modern concepts such as the worker-pool and producer/consumer programming patterns. While PaPy is suitable for pipelines concerned with data-processing and analysis (data *reduction*), it also could be useful for replicated simulations and other types of workflows which involve computationally-expensive components that generate large volumes of data.

## List of abbreviations

API: application programming interface; DAG: directed acyclic graph; FBP: flow-based programming; IPC: inter-process communication; MD: molecular dynamics; RPyC: remote Python calls; shm: shared memory; WMS: workflow management system

## Authors' contributions

MC wrote PaPy; MC and CM tested the code and wrote the paper. All authors read and approved the final manuscript.

## Availability and requirements

• **Project name**: PaPy

• **Project homepage**: http://muralab.org/PaPy

• **Operating system**: GNU/Linux

• **Programming language**: Python

• **Other requirements**: A modern release of Python (≥2.5) is advised; the standard, freely-available Python package RPyC is an optional dependency (for distributed computing).

• **License**: New BSD License

• **Any restrictions to use by non-academics**: None; the software is readily available to anyone wishing to use it.

## Supplementary Material

Additional file 1 **This supplementary file provides the following material, along with complete and fully annotated source-code for each example: (§1)**. Two simple examples of workflows (useful as pipeline templates), one showing a generic *forked *pipeline and the other focusing on the usage of NuBio; (§2) A detailed description of our more complicated case-study (simulation-based refinement of homology model loops); (§3) Further notes on PaPy's platform independence, as well as the relationship between the Dagger and Plumber classes; (§4) A brief overview of PaPy's scope and implementation, in relation to a fully-integrated WMS suite(KNIME). Click here for file
