# Supplementary material for: A lightweight, flow-based toolkit for parallel and distributed bioinformatics pipelines
Source: BMC Bioinformatics. 2011 Feb 25;12:61. doi: 10.1186/1471-2105-12-61 (PMC3051902; doi:10.1186/1471-2105-12-61)
Supplement: Additional file 1 — This supplementary file provides the following material, along with complete and fully annotated source-code for each example: (§1). Two simple examples of workflows (useful as pipeline templates), one showing a generic forked pipeline and the other focusing on the usage of NuBio; (§2) A detailed description of our more complicated case-study (simulation-based refinement of homology model loops); (§3) Further notes on PaPy's platform independence, as well as the relationship between the Dagger and Plumber classes; (§4) A brief overview of PaPy's scope and implementation, in relation to a fully-integrated WMS suite(KNIME). [file 1471-2105-12-61-S1.PDF]

Additional File for

A Lightweight, Flow-based Toolkit for Parallel and Distributed Bioinformatics Pipelines

Marcin Cieślik[1,2] & Cameron Mura[1,2]*

[1]Department of Chemistry, University of Virginia, Charlottesville, VA 22904-4319; [2]Structural, Computational Biology & Biophysics, University of Virginia Health Sciences, Charlottesville, VA 22908 USA; *cmura@virginia.edu

19 December 2010

Contents

1 Some simple, generic examples **1**
1.1 A forked pipeline 2
1.2 A sample workflow using the NuBio package 4

2 An intricate example: Simulation-based loop refinement **5**
2.1 Overview 5
2.2 Detailed description 5
2.2.1 Structure of the code 6
2.2.2 Functions, workers, pipers 7
2.2.3 Pipes and branches 7
2.2.4 Parallel processing 7
2.3 Python code for this workflow 8

3 Some further notes on PaPy **14**
3.1 Platform-independence and installation 14
3.2 The Dagger and Plumber classes 14
3.3 PaPy and cloud computing 15

4 A comparative overview of PaPy and a heavyweight WMS suite (KNIME) **15**

References **17**

1 Generic pipeline examples in PaPy

To illustrate the general use of PaPy as a toolkit for workflow design, construction, and execution, several examples of common pipeline patterns (simple forks/joins, produce/spawn/consume, *etc.*) are included in

the ‘`doc/examples`’ directory of the source-code distribution. The following subsections present Python code for two typical scenarios: A generic fork/join pipeline (§1.1), and a workflow that illustrates the incorporation of the NuBio package (§1.2). Both code samples are annotated with descriptive comments, particularly as regards segments that are specific to PaPy and its usage.

1.1 Prototype for a forked pipeline

The following code for this generic example can also be found in the PaPy source as ‘`doc/workflows/pipeline.py`’:

```
#!/usr/bin/env python
# -*- coding: utf-8 -*-
"""
This file (doc/workflows/pipeline.py in the PaPy src) provides a prototype of a
pipeline; use it as a starting-point to construct your own pipelines. The
construction of a pipeline can be split into distinct parts, for several
reasons. Most importantly, this makes the logic of your code more modular, as
it detaches the definition of a workflow from the actual runtime (i.e. the real
data and computational resources to solve the particular instance of the
problem at hand). Furthermore, this approach simplifies workflow execution and
record-keeping (provenance) by allowing one to group all the necessary elements
into a single executable script file.

All steps in the following workflow are as explicit as possible. If you prefer
to use a less flexible (but more compact and implicit) approach, via the API
features, then please refer to the documentation.

 xrange
   |
  fork (linear)
 /    \
L      R (parallel, signle shared resource)
 \    /
  join (linear)
    |
  print

"""

#------------------------------------------------------------------------------
# Part 0: Import the PaPy infrastructure.
# interface of the API:
from papy import Plumber, Piper, Worker
from papy.util.func import print_
# NuMap provides parallel/distributed worker-pool functionality and the
# ’imports’ wrapper:
from numap import NuMap, imports
# logging support:
import logging
from papy.util.config import start_logger
start_logger(log_to_stream=True, log_to_stream_level=logging.ERROR)

#------------------------------------------------------------------------------
# Part 1: Define user functions
def fork(inbox):
    msg = inbox[0]
    return msg

@imports([’socket’, ’os’, ’threading’])
def who_am_i(inbox):
    """
    This function identifies the host process.
    """
    return "process:%s" % os.getpid()

```

```
def join(inbox):
    left_branch, right_branch = inbox
    return "joined result from %s and %s" % (left_branch, right_branch)


#----------------------------------------------------------------------------
# Part 2: Define the topology
def pipeline(resource):
    # initialize Worker instances (i.e. wrap the functions).
    w_fork = Worker(fork)
    w_who_am_i = Worker(who_am_i)
    w_join = Worker(join)
    # initialize Piper instances (i.e. attach functions to runtime)
    p_fork = Piper(w_fork, name='Fork')
    L = Piper(w_who_am_i, parallel=resource, branch=1)
    R = Piper(w_who_am_i, parallel=resource, branch=2)
    p_join = Piper(w_join, name='Join')
    p_print = Piper(print_)
    # create the pipeline and connect pipers
    workflow = Plumber()
    workflow.add_pipe((p_fork, L))
    workflow.add_pipe((p_fork, R))
    workflow.add_pipe((L, p_join))
    workflow.add_pipe((R, p_join))
    workflow.add_pipe((p_join, p_print))
    return workflow

#----------------------------------------------------------------------------
# Part 3: Parse the arguments
def options(args):
    size = int(args.get('--size', 10))
    worker_num = int(args.get('--worker_num', 4))
    return (size, worker_num)

#----------------------------------------------------------------------------
# Part 4: Define the compute resources
def resources(args):
    size, worker_num = args
    rsrc = NuMap(worker_num=worker_num)
    return rsrc

#----------------------------------------------------------------------------
# Part 5: Create the input data
def data(args):
    size, worker_num = args
    return xrange(size)

#----------------------------------------------------------------------------
# Part 6: Execute
if __name__ == '__main__':
    # get command-line arguments using getopt
    import sys
    from getopt import getopt
    args = dict(getopt(sys.argv[1:], '', ['size=', 'worker_num='])[0])
    # parse options
    opts = options(args)
    # definie/initialize resources
    rsrc = resources(opts)
    # define/create input data
    inpt = data(opts)
    # attach resources to pipeline
    workflow = pipeline(rsrc)
    # connect and start pipeline
    workflow.start([inpt])
    # run and wait until pipeline is finished
    workflow.run()
    workflow.wait()
    workflow.pause()
```

```
    workflow.stop()
    print "Runtime: %.2fs" % workflow.stats['run_time']
```

1.2 A simple, specific workflow using NuBio

The code for the following example can be found in the PaPy source as '`doc/examples/hello_workflow.py`':

```
#!/usr/bin/env python
# -*- coding: utf-8 -*-

#------------------------------------------------------------------------------
# Step 0 (importing the library)
from numap import NuMap, imports
from papy.core import Worker, Piper, Plumber
from nubio import AaSeq, NtSeq
# For optional logging functionality:
# from papy.util.config import start_logger
# Log to both session stream and file (default name 'PaPy_log', to which
# results will be appended):
# start_logger(log_to_file=True, log_to_stream=True)


#------------------------------------------------------------------------------
# Step 1 (writing a worker function)
# clean_Seq takes a raw sequence, creates an array object of amino- or
# nucleic- acid and replaces certain characters. It can be used as follows
# >>> arr = clean_seq(['AGA.TA'], type='aa', fixes=[('.', '-')])
# >>> print arr
def clean_seq(inbox, type, fixes):
    seq = inbox[0]
    if type == 'aa':
        arr = AaSeq(seq)
    elif type == 'nt':
        arr = NtSeq(seq)
    else:
        raise ValueError("unknow sequence type %s" % type)
    for bad, good in fixes:
        arr.str('replace', bad, good)
    return arr

# timestamp take an array object and annotates it with the current data.
# the function depends on ``time`` module, attached to the function
# definition via the ``imports`` decorator. It is simply called:
# >>> arr = timesamp([arr])
# >>> print arr.meta['timestamp']
@imports(['time'])
def timestamp(inbox):
    arr = inbox[0]
    arr.meta['timestamp'] = "%s_%s_%s@%s:%s:%s" % time.localtime()[0:6]
    return arr

#------------------------------------------------------------------------------
# Step 2 (wrapping function into workers)
# wraps clean_seq and defines a specific sequence type and fixes
cleaner = Worker(clean_seq, kwargs={'type':'aa', 'fixes':[('.', '-')]})
# >>> arr = cleaner(['AGA.TA'])
# wraps timestamp
stamper = Worker(timestamp)
# >>> arr = stamper([arr])

#------------------------------------------------------------------------------
# Step 3 (representing computational resources)
# creates a resource that allows to utilize all local processors
local_computer = NuMap()
```

```

#------------------------------------------------------------------------------
# Step 4 (creating processing nodes)
# this attaches a single computational resource to the two processing nodes
# the stamper_node will be tracked i.e. it will store the results of computation
# in memory.
cleaner_node = Piper(cleaner, parallel=local_computer)
stamper_node = Piper(stamper, parallel=local_computer, track=True)

#------------------------------------------------------------------------------
# Step 5 (constructing a workflow graph)
# we construct a workflow graph add the two processing nodes and define the
# connection between them.
workflow = Plumber()
workflow.add_pipe((cleaner_node, stamper_node))

#------------------------------------------------------------------------------
# Step 6 (execute the workflow)
# this starts the workflow, processes data in the "background" and waits
# until all data-items have been processed.
workflow.start([['AGA.TA', 'TG..AA']])
workflow.run()
workflow.wait()
results = workflow.stats['pipers_tracked'][stamper_node][0]
for seq in results.values():
    print "Object \"%s\" has time stamp: %s " % (seq, seq.meta['timestamp'])
```

2 An intricate example: Simulation-based loop refinement

2.1 Overview of this workflow

Using simulation-based refinement of homology model loops as an example from structural bioinformatics, this use-case shows that rather intricate workflows can be expressed as PaPy pipelines. In particular, this workflow processes batches of homology models and attempts to refine the loop structures *via* successive stages of energy minimization, equilibration, and then brief molecular dynamics (MD) simulations. As mentioned in the main text, this workflow was implemented not to address the specific scientific problem of loop refinement (there are alternative, potentially better approaches; see (1) and references therein for a recent study in this area), but rather to illustrate the usage of PaPy in constructing and enacting an intricate, multi-stage, parallelized workflow.

2.2 A walk through the steps in this workflow

This workflow provides a concrete example of several PaPy-related concepts, including *(i)* Data import and parsing (the input is a ModBase XML file specifying the starting homology models); *(ii)* the *function*/Worker/Piper relationship (Fig. 1 of the text), including chaining functions into a single composite Worker; *(iii)* multiple streams of data-flow (see the branch points in Fig. 5 of the main text); *(iv)* implementation of common workflow patterns, such as the *produce*/*spawn*/*consume* idiom (piper-6, layer-7, piper-8 in Fig. 5); and *(v)* integration of external software packages, such as STRIDE (2) and MMTK (3), into a pipeline.

This case-study is lengthy and dense (§2.3 below), but the modular design of the pipeline code makes it more interpretable, without obscuring the underlying workflow logic. This pipeline processes batches of homology models of the Sm-like protein ‘Hfq’, downloaded from a database of comparative structural models (MODBASE; (4)). The overall workflow (Fig. 5) refines the 3D model structures *via* successive steps of *(i)* minimization/equilibration of the starting structure for each model, followed by *(ii)* multiple,

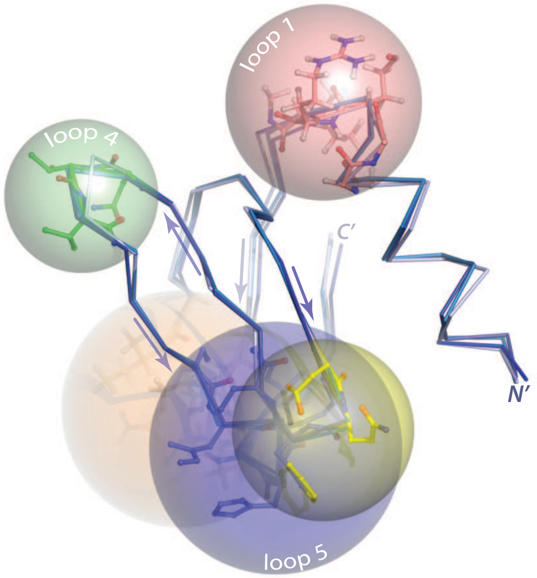

Figure S1: Backbone C_α traces are shown for one of the LSm homology models (Q6F9W2) used in this case-study; for clarity, the *N*- and *C*-termini are labelled, and arrows denote local strand directionality. Successive stages of refinement are indicated as blue hues ramped from dark (initial structure) to medium (minimization, equilibration) to light (refined model). The MD_n^{loop} systems (Fig. 5), comprised of loop residues and neighbors, are indicated by bounding spheres centered on the characteristic β-turn loops 1 (red), 2 (orange), 3 (yellow), 4 (green), and 5 (blue) of the ≈70-residue LSm fold; these subsystems are *produced* in the middle of the workflow (Fig. 5), *spawn* short MD simulations, and are then *consumed* in the '`combine_loop_models`' reduction step.

independent MD simulations, run in parallel for each separate loop of each model. As shown in the workflow schematic (Fig. 5), the equilibrate_model processing node consists of chained workers; the first worker performs *in vacuo* potential energy minimization, followed by a worker specifying thermalization and short dynamics runs at 300 K. One downstream fork of the model equilibration node then calls STRIDE to assign secondary structure labels to residues (based on backbone ϕ-ψ values), and individual loops are identified on-the-fly, as contiguous stretches of residues that are neither α-helical nor β-sheet. In the next set of steps, each loop of the pre-equilibrated model is then refined, in parallel (the spawned 'MD_n' pipers in Fig. 5). Using an algorithm written specifically for this use-case (see the `call_stride`, `define_loops`, `create_loop_models` steps near lines 110-220 of the code), the equilibrated starting model is partitioned into (possibly overlapping) spherical regions centered at each loop (Fig. S1 above). Each such region contains all residues comprising the loop, in addition to surrounding residues with which the loop might interact; this bounding sphere is padded with a 5 Å cushion, as set by '`sphere_margin`' in the '`w_create_loop_models`' worker (near line 300). The positions of other residues (distal to the loop) are fixed during the short MD simulations. For a model with *n* loops, the *n* loop-refined structures are then merged, together with the non-loop region, in the final stages of the workflow (pipers 8 and 9 in Fig. 5); in this last step, final coordinates are obtained by updating the atomic positions of all loop residues in the equilibrated model with the configurations from the per-loop MD simulations.

2.2.1 Structure of the code

This sample pipeline highlights several features of PaPy. First and foremost, it demonstrates PaPy's role as a toolkit for the design and execution of parallel and distributed pipelines *in the context of Python*. PaPy pipelines are expressed as Python scripts that simply call upon the 'papy' module (see, *e.g.*, '`from papy import ...`' statements). Though there are no fixed rules or constraints on how Python code should be structured, a modular design leads to cleaner, re-usable code that is more easily maintained and interpreted by others. As is the case with many algorithms, a natural feature of workflows is that various aspects of the code are independent and logically separable – low-level function definitions can be insulated from higher-level pipeline components (specification of pipeline topology, definition of worker-pools), and this level can be shielded from overall details of workflow execution/monitoring/logging. This natural decoupling between function code, workflow topology, and parameters/compute resources guided the design of the

loop-refinement pipeline: In the code shown in §2.3, the basic functionality (*e.g.*, computing loop boundaries) is defined in the large Stage 1 block (lines ≈25-270), while Stage 2 assembles pipers and workers into a specific pipeline topology (lines ≈270-360), and Stage 3 executes the workflow.

2.2.2 Functions, workers, and pipers

Together with a problem-specific set of parameters (passed as arguments), the functionality defined in Stage 1 is used to create callable objects (Workers) that define the low-level activities of the workflow. This low-level behavior is encapsulated as pipeline-specific workers (see the segment of Worker definitions near lines 270-318), and is combined in Stage 2 with parallel worker-pool definitions (NuMap objects) to create pipers (see the Piper instantiations near lines 320-335). The pipeline topology, and therefore pattern of data-flow, is defined in Stage 2 by the interconnections between Piper nodes (see §2.2.3 below).

As previously noted, Workers and their wrapped functions can be flexibly defined in PaPy: The Stage 1 procedures may correspond to nothing more than ordinary, user-defined Python functions (*e.g.*, '`create_dummy_files`' near line 30). Alternatively, external Python libraries can be used (*e.g.*, the MMTK-wrapping '`create_model`' near line 45), as can third-party, non-Python executables (*e.g.*, the STRIDE-based '`call_stride`' near line 110) or web-accessible data/services. The first argument to these functions is a Python tuple, containing the results from all incoming pipes when the function is to be used in a workflow. The (optional) imports decorator can be used to attach Python '`import`' statements to function code, thereby allowing the function to be evaluated remotely (Python modules required by the imported function also must be present on the remote host). Finally, as an example of the strategy of composing functions to collapse a pipeline segment into a single node and avoid unnecessary IPC (Fig. 1), note that the energy minimization and thermal equilibration operations (defined as separate workers near lines 280 and 290, respectively) are composed into a single Worker instance ('`w_minimize_equilibrate_model`') near line 300; this is also indicated schematically in Fig. 5.

2.2.3 Pipes and branches

Data-flow through the pipeline is literally defined by creating pipes between pairs or sequences of nodes, as shown by the '`.add_pipe`' method invocations near lines 340-360. Note that the pipeline has a branch point after creation of an equilibrated 3D model (piper 3 in Fig. 5) and two merge points (at pipers 6 and 9). The two mergings correspond to *(i)* creation of spherical loop regions (Fig. 5, green and black arrows feeding into piper 6) and *(ii)* assembly of the final refined model (Fig. 5, gray and black arrows into piper 9). In general, a branch might be used to propagate data directly between functions in different places of a pipeline, or to carry-out sub-tasks. In the present loop-refinement workflow, a sample 'sub-task' is identification of the loops in a protein, based on secondary structure assignments computed by STRIDE.

2.2.4 Parallel processing

Ideally, a workflow would exhibit efficient execution on datasets of ever-increasing scale (scalability); this issue becomes especially acute with CPU-intensive methods, such as atomistic simulations or *omics*-scale datasets. Parallel evaluation offers an approach to handling larger-scale datasets and, depending on the degree of coupling between individual data-items, may occur at the level of a pipeline node or at the level of the overall workflow topology. An example of the former (node-level) would be an individual MD worker node executing in multi-threaded manner over many cores, and an example of the latter (workflow-level) would be produce/spawn/consume to distribute calculations. A PaPy workflow can implement different varieties of parallel evaluation at the node- or pipeline-level, including local/remote and multi-threaded/multi-processor parallelism (Fig. 1B). The NuMap facility supplies PaPy with a unified interface for handling worker-pools

(parallel or not), and therefore provides a common mechanism for handling parallelism and assigning computational resources to processing nodes. By default, NuMap instances utilize all locally-available CPUs. For the loop-refinement workflow, a ‘`local_computer`’ worker-pool resource is defined (near line 270) as a 2-worker NuMap instance, using a relatively high value of 100 for the ‘buffer’ argument. (The buffer sets an upper-limit on the number of memory-buffered elements [input and pending results], as further described in the documentation for the NuMap class.) The most computationally-expensive nodes in the loop-refinement workflow – equilibration (piper-3 in Fig. 5, line 324) and MD simulations (pipers 7.*n* in Fig. 5, line 330) – are assigned to the parallel pool.

In conclusion, note that loop-refinement, as a PaPy pipeline, is parallelizable at several simultaneous levels: *(i)* Processing of (independent) input homology models is trivially parallel. *(ii)* A single data-item (a given homology model traversing the pipeline) is parallelized by partitioning it into discrete 3D spatial regions, spawning multiple short MD runs centered on each loop in turn. *(iii)* Depending on the capabilities of the simulation code, numerical integration of MD trajectories for each such loop can be performed in a highly-efficient, parallel manner. As illustrated by the present workflow, parallel processing at levels *(i)* and *(ii)* can be achieved *via* PaPy.

2.3 Python code for this workflow

The following code was tested on Linux workstations (Fedora and Gentoo distributions) with recent stable releases of Python (2.6.5) and MMTK (2.7.2).

```
1 #!/usr/bin/env python
2 """
3 """
4 #----------------------------------------------------------------------------
5 # Stage 0: Import PaPy and related (NuMap) infrastructure; see online docs
6 # for complete description of the API.
7 
8 # papy and numap modules:
9 from papy.core import Worker, Piper, Plumber
10 # the parallel NuMap worker-pool functionality and imports wrapper:
11 from numap import NuMap, imports
12 
13 # logging support
14 from logging import getLogger
15 from papy.util.config import start_logger, get_defaults
16 start_logger(log_to_file=False, log_to_stream=True)
17 # following version is less noisy (no logging to stream):
18 # start_logger(log_to_file=False, log_to_stream=False)
19 
20 import sys
21 sys.stderr = open(’/dev/null’, ’w’)
22 LOOP_NUM = 10   # maximum number of loops to consider in homology models
23 
24 
25 #----------------------------------------------------------------------------
26 # Stage 1: Define the functions that dictate the low-level functionality of the
27 # workflow
28 @imports([’re’, ’StringIO’])
29 def create_dummy_files(input_file):
30     handle = open(input_file)
31     match_content = re.compile(’<content>(.*?)</content>.*?UP (.*?)\s+\d’, re.DOTALL)
32     file_strings = match_content.finditer(handle.read())
33     model = 0
34     while True:
35         file_content, model_name = file_strings.next().groups()
36         yield (StringIO.StringIO(file_content), model_name)
37         model += 1
38         # To do only first 2 models (not all of them):
39         if model == 2:
40             raise StopIteration
```

```

@imports(['MMTK', 'MMTK.PDB', 'MMTK.Proteins', 'MMTK.ForceFields'])
def create_model(inbox, forcefield, save_file):
    dummy_file, model_name = inbox[0]
    print 'create_model: %s' % model_name
    # create the protein
    configuration = MMTK.PDB.PDBConfiguration(dummy_file)
    chains = configuration.createPeptideChains()
    protein = MMTK.Proteins.Protein(chains)
    # create the forcefield
    if forcefield == 'amber94':
        forcefield = MMTK.ForceFields.Amber94ForceField()
    elif forcefield == 'amber99':
        forcefield = MMTK.ForceFields.Amber99ForceField()
    # create and fill the universe
    universe = MMTK.InfiniteUniverse(name=model_name)
    universe.setForceField(forcefield)
    universe.protein = protein
    # ...and, optionally, write-out the initial coordinates to a PDB file:
    if save_file:
        universe.protein.writeToFile('results/%s_initial.pdb' % universe.name)
    return universe

@imports(['MMTK.Trajectory', 'MMTK.Minimization'])
def minimize_model(inbox, steps, convergence, save_file, save_log):
    universe = inbox[0]
    print 'minimize_model: %s' % universe.name
    actions = []
    if save_log:
        actions.append(
         MMTK.Trajectory.LogOutput('results/%s_minimization.log' % universe.name))
    minimizer = MMTK.Minimization.ConjugateGradientMinimizer(universe, actions=actions)
    minimizer(convergence=convergence, steps=steps)
    if save_file:
        universe.protein.writeToFile('results/%s_minimized.pdb' % universe.name)
    return universe

@imports(['MMTK', 'MMTK.Dynamics', 'MMTK.Trajectory'])
def equilibrate_model(inbox, steps, T_start, T_stop, T_step, save_file, save_log):
    # 'T' refers to Temperature (in K)
    universe = inbox[0]
    print 'equilibrate_model: %s' % universe.name
    universe.initializeVelocitiesToTemperature(T_start * MMTK.Units.K)
    integrator = MMTK.Dynamics.VelocityVerletIntegrator(universe,
                                       delta_t=1. * MMTK.Units.fs)
    actions = [
        # Heat from T_start K to T_stop K, applying a temperature
        # change of T_step K/fs; scale velocities at every step.
        MMTK.Dynamics.Heater(T_start * MMTK.Units.K,
                             T_stop * MMTK.Units.K,
                             T_step * MMTK.Units.K / MMTK.Units.fs,
                             0, None, 1),
        # Remove global translation every 50 steps.
        MMTK.Dynamics.TranslationRemover(0, None, 50),
        # Remove global rotation every 50 steps.
        MMTK.Dynamics.RotationRemover(0, None, 50)]
    if save_log:
        # log output to file:
        actions.append(MMTK.Trajectory.LogOutput(
            'results/%s_equilibration.log' % universe.name))
    # execute it:
    integrator(steps=steps, actions=actions)
    if save_file:
        universe.protein.writeToFile(
            'results/%s_equilibrated.pdb' % universe.name)
    return universe

@imports(['subprocess'])
```

```
def call_stride(inbox):
    # Using the program 'Stride' for loop determination...
    universe = inbox[0]
    print 'call_stride on model: %s' % universe.name
    filename = "results/%s_equilibrated.pdb" % universe.name
    process = subprocess.Popen('stride %s' % filename, shell=True,
                               stdin=subprocess.PIPE,
                               stdout=subprocess.PIPE)
    # Parsing Stride-specific output (e.g. the following 'ASG' business):
    output = []
    for line in process.stdout.xreadlines():
        if line.startswith('ASG'):
            res_name = line[5:8] # we use 3
            chain_id = line[9]
            if chain_id == '-': # stride ' ' -> '-' rename
                chain_id = ' '
            try:
                res_id = int(float(line[10:15]))
                res_ic = ' '
            except ValueError:
                res_id = float(line[10:14])
                res_ic = line[14]
            ss_code = line[24]
            phi = float(line[43:49])
            psi = float(line[53:59])
            asa = float(line[62:69])
            output.append((res_id, ss_code))
            # output[(chain_id, (res_name, res_id, res_ic))] =
            # (ss_code, phi, psi, asa)
    if not output:
        # this propably means a Calpha only chain has been supplied
        raise RuntimeError
    return output

def define_loops(inbox, min_size, max_gaps):
    print 'define loops'
    stride_results = inbox[0]
    loops = []
    new = True
    for res_id, ss_code in stride_results:
        if ss_code in ('E', 'H'):
            new = True
            continue
        else:
            if new:
                loops.append([])
            new = False
            loops[-1].append(res_id)
    return loops

@imports(['MMTK', 'MMTK.Proteins', 'MMTK.PDB', 'os'])
def create_loop_models(inbox, loop_num, sphere_margin, save_file):
    # This function does much of the heavy-lifting that is specific to this
    # pipeline (i.e., it creates the loop models...).
    loops, universe = inbox
    print 'create loop models: %s' % universe.name, loops
    residues = universe.protein.residues()
    loop_models = []
    try:
        for i, loop in enumerate(loops):
            # Determine which residues belong to a loop, grouping them as an
            # MMTK 'Collection':
            loop_res = MMTK.Collections.Collection()
            for res_id in loop:
                loop_res.addObject(residues[res_id - 1])
            # Determine a bounding sphere for the residues:
            bs = loop_res.boundingSphere()
            # select all residues in protein within the sphere plus margin
```

```
            loop_sphere = residues.selectShell(bs.center, bs.radius + sphere_margin)
            # Determine the offset of the loop
            offset_protein = loop[0] - 1
            offset_loop = list(loop_sphere).index(residues[offset_protein])
            #### Create new universe
            # save the sphere around the loop as a new file
            loop_name = "%s_loop%s" % (universe.name, i)
            loop_file = 'results/%s_equilibrated.pdb' % loop_name
            pdb_file = MMTK.PDB.PDBOutputFile(loop_file)
            pdb_file.write(loop_sphere)
            pdb_file.close()
            # load the new file as if it was a proper chain (the loop is proper)
            # MMTK writes terminal forms of residues
            configuration = MMTK.PDB.PDBConfiguration(loop_file)
            chain = MMTK.Proteins.PeptideChain(configuration.peptide_chains[0],
                                  #n_terminus=(offset_protein == 0),
                                  n_terminus=loop_sphere[0] == residues[0],
                                  c_terminus=loop_sphere[-1] == residues[-1])
            protein = MMTK.Proteins.Protein(chain)
            loop_universe = MMTK.InfiniteUniverse(name=loop_name)
            loop_universe.setForceField(universe.forcefield())
            loop_universe.protein = protein
            loop_residues = loop_universe.protein.residues()
            # now fix all atoms which are not the initial loop
            # select real loop residues
            left_residues = loop_residues[0:offset_loop]
            right_residues = loop_residues[offset_loop + len(loop):]
            #print 'loop: %s' % (i + 1,)
            #print 'residues in loop: %s' % list(residues[offset_protein:offset_protein + len(loop)])
            #print 'residues in shell: %s' % list(loop_sphere)
            #print 'residues to be fixed: %s' % (left_residues + right_residues)
            for residue in left_residues + right_residues:
                for atom in residue.atomList():
                    atom.fixed = True
            loop_models.append((loop_universe, (offset_loop, offset_protein, len(loop))))
            if not save_file:
                os.unlink(loop_file)
    except Exception, e:
        print i, loop, list(loop_sphere), e
        raise
    print 'created loop models: %s' % loop_models
    for i in xrange(loop_num - len(loop_models)):
        loop_models.append(None)
    return loop_models

@imports(['MMTK', 'MMTK.Dynamics', 'MMTK.Trajectory'])
def md_loop_model(inbox, steps, temp, save_file, save_trajectory, save_log):
    # The following is for produce/spawn/consume-related padding:
    if inbox[0] is None:
        return None
    loop_universe = inbox[0][0]
    print 'md of loop model: %s' % loop_universe.name
    actions = []
    if save_log:
        actions.append(MMTK.Trajectory.LogOutput('results/%s_refinement.log' % \
                                            loop_universe.name))
    if save_trajectory:
        traj = MMTK.Trajectory.Trajectory(loop_universe, "%s.nc" % loop_universe.name, "w")
        # Write every second step to the trajectory file.
        actions.append(MMTK.Trajectory.TrajectoryOutput(traj, \
                        ("time", "energy", "thermodynamic", "configuration"),
                        0, None, 2))

    loop_universe.initializeVelocitiesToTemperature(temp * MMTK.Units.K)
    integrator = MMTK.Dynamics.VelocityVerletIntegrator(loop_universe, delta_t=1. * MMTK.Units.fs)
    integrator(steps=steps, actions=actions)
    if save_trajectory:
        traj.close()
```

```
    if save_file:
        loop_universe.protein.writeToFile('results/%s_refined.pdb' % loop_universe.name)
    return inbox[0]

def combine_loop_models(inboxes):
    print 'collect loop models model'
    loop_universes_offsets = [i[0] for i in inboxes if i[0] is not None]
    return loop_universes_offsets

@imports(['itertools'])
def make_refined_model(inbox, save_file):
    combined, initial = inbox
    print 'make refined model: %s' % initial.name
    residues = initial.protein[0].residues() # residues in the first peptide chain
    for loop_universe, (offset_loop, offset_protein, len_loop) in combined:
        initial_residues = residues[offset_protein:offset_protein + len_loop]
        refined_residues = loop_universe.protein[0].residues()[offset_loop:offset_loop + len_loop]
        for ir, rr in itertools.izip(initial_residues, refined_residues):
            for ia, ra in itertools.izip(ir.atomList(), rr.atomList()):
                ia.setPosition(ra.position())
    if save_file:
        initial.protein.writeToFile('results/%s_refined.pdb' % initial.name)


#-------------------------------------------------------------------------------
# Stage 2: Define the workflow's Workers and Pipers
def pipeline():
    local_computer = NuMap(worker_num=2, buffer=100)
    pipes = Plumber()
    # initialize Worker instances (i.e. wrap the functions)...
    w_create_model = Worker(create_model, kwargs={
                                              'forcefield': 'amber99',
                                              'save_file':True
                                              })
    # 100 steps of minimization found to be enough for convergence:
    w_minimize_model = Worker(minimize_model, kwargs={
                                                  'steps': 150,
                                                  'convergence':1.0e-4,
                                                  'save_log':True,
                                                  'save_file':True
                                                  })
    w_equilibrate_model = Worker(equilibrate_model, kwargs={
                                                        'steps':250,
                                                        'T_start':50., # K
                                                        'T_stop':300., # K
                                                        'T_step':0.5,  # K
                                                        'save_log':True,
                                                        'save_file':True
                                                        })
    # Composite worker:
    w_minimize_equilibrate_model = Worker((w_minimize_model, w_equilibrate_model))
    w_call_stride = Worker(call_stride)
    w_define_loops = Worker(define_loops, kwargs={
                                              'min_size':7,
                                              'max_gaps':2
                                              })
    w_create_loop_models = Worker(create_loop_models, kwargs={
                                                          'sphere_margin':0.5, # nm
                                                          'loop_num':LOOP_NUM,
                                                          'save_file':True
                                                          })
    # 50000 steps (= 50 ps dynamics) at 300 K:
    w_md_loop_model = Worker(md_loop_model, kwargs={
                                                'steps':50000,
                                                'temp':300, # K
                                                'save_file':True,
                                                'save_trajectory':False,
                                                'save_log':True
```

```
                                                    })
    w_combine_loop_models = Worker(combine_loop_models)
    w_make_refined_model = Worker(make_refined_model, kwargs={
                                                               'save_file':True
                                                               })

    # initialize Piper instances (i.e. attach functions to runtime)
    p_create_model = Piper(w_create_model, debug=True)
    # p_minimize_model = Piper(w_minimize_model, parallel=local_computer,
    #                          debug=True)
    p_equilibrate_model = Piper(w_minimize_equilibrate_model,
                                parallel=local_computer, debug=True)
    P_call_stride = Piper(w_call_stride, debug=True)
    p_define_loops = Piper(w_define_loops, debug=True)
    p_create_loop_models = Piper(w_create_loop_models,
                                 debug=False, produce=LOOP_NUM)
    p_md_loop_model = Piper(w_md_loop_model, debug=True,
                            parallel=local_computer, spawn=LOOP_NUM)
    p_combine_loop_models = Piper(w_combine_loop_models, debug=True,
                                  consume=LOOP_NUM)
    p_make_refined_model = Piper(w_make_refined_model, debug=True)

    # Create the pipeline and connect pipers:

    # Central pipeline (pipers 1 -> 2 -> 3 -> 6 -> layer-7 -> 8 -> 9 -> 10 in
    # Figure 5 of the manuscript; ---{6 -> layer-7 -> 8}--- is the produce/
    # spawn/consume part of the workflow):
    pipes.add_pipe((
                    p_create_model,
                    #p_minimize_model,
                    p_equilibrate_model,
                    p_create_loop_models,
                    p_md_loop_model,
                    p_combine_loop_models,
                    p_make_refined_model
                    ))
    # The 'Stride' branch of the pipeline (pipers 4, 5 in the manuscript):
    pipes.add_pipe((
                    p_equilibrate_model,
                    P_call_stride,
                    p_define_loops,
                    p_create_loop_models
                    ))
    # A 'short-circuit' of the pipeline, jumping from piper 3 --> 9 (i.e.,
    # no loop refinement, just general equilibration):
    pipes.add_pipe((
                    p_equilibrate_model,
                    p_make_refined_model
                    ))
    return pipes


#-------------------------------------------------------------------------------
# Stage 3: Execute the pipeline
if __name__ == '__main__':
    pipes = pipeline()
    pipes.start([create_dummy_files('data/hfq_models.xml')])
    pipes.run()
    pipes.wait()
    pipes.pause()
    pipes.stop()
    print pipes.stats
```

3 Some further notes on PaPy

3.1 Platform-independence and installation

The PaPy codebase and its auxiliary toolkits (NuMap, NuBio) were written in CPython (version 2.6), which is the standard/default, cross-platform implementation of Python. In much the same way as PyMOL or other Python-based software packages, PaPy derives its platform-independence from the facts that *(i)* Python itself is platform-independent and *(ii)* PaPy is written in pure Python (*i.e.*, no special tricks, customizations, add-on modules, or other dependencies were introduced beyond the defintion of the core Python language). Thus, although PaPy was developed and tested on several variants of Linux (Ubuntu, Gentoo, Fedora, Arch), the software can in principle be used on any Python-capable platform (Unix/Linux, Mac, Windows operating systems); assuming the root cause was PaPy (and not Python-related), we would be happy to address any incompatibilites discovered by users on non-Linux platforms.

Detailed installation scenarios – ranging from simple to advanced – are provided in the PaPy manual, including step-by-step instructions for three common Linux distributions (Gentoo, Ubuntu, Fedora). PaPy is most easily obtained and installed *via* the Python Package Index (PyPI), using 'setuptools' and the simple command-line invocation '`easy_install papy`'. The PaPy manual also describes a more advanced installation scenario, using the Python 'virtual environments' facility in order to install PaPy into an insulated environment, rather than system-wide.

In terms of platform-independence and more advanced usage of PaPy, it is worth noting that direct communication between pipers (to avoid interprocess communication overhead) comes at the cost of platform-independence, as the operating system must properly support the chosen transmission mechanism. For instance, communication between processes on a single host *via* Unix pipes is necessarily restricted to Unix/Linux systems. This stems from an intrinsic, well-known constraint in software engineering: platform–(in)dependence and advanced functionality are features of a software system that generally counteract, and therefore limit, one another. PaPy provides platform-independent alternatives for any such platform-specific feature (see, *e.g.*, Table 3 of the main text for alternatives to Unix pipes).

3.2 An additional note on the Dagger and Plumber classes

PaPy's Dagger and Plumber classes are core components (Table 2 of the main text) that are conceptually quite closely related, but that also subtly differ in terms of both low-level (software) implementation and user-exposed functionality. Briefly, Plumber objects can be viewed as special Dagger objects with extended functionality: A specific Dagger instance defines a PaPy pipeline/workflow in terms of connections between nodes (Piper objects), but exactly the same workflow (*i.e.* same nodes, same topology of interconnections between nodes) can also be constructed as a Plumber object, with the benefit that the Plumber class endows the pipeline object with methods for user interaction. Derived from PaPy's even lower-level 'DictGraph' class (a dictionary-based data structure for representing arbitrary graphs), the Dagger class provides the basic methods for adding, deleting, and connecting Piper instances and 'pipes' (edges that links two specific Piper nodes). The Plumber is a subclass of the Dagger class that inherits methods from Dagger objects and can be used as a Dagger, but that also extends the functionality of the Dagger class by adding methods for higher-level (run-time) workflow manipulation and interaction – *e.g.*, loading/saving workflows or starting/stopping/pausing a pipeline. (As detailed in the API, these methods are defined with intuitively obvious names, such as '`.stop()`'.)

More specific descriptions of the low-level definitions of the Dagger and Plumber classes can be found in the PaPy manual (pgs 18-21). Both Dagger and Plumber are classes within the higher-level papy.core module, and a comparison of their API entries (see Manual) reveals the subtle differences in the properties and capabilities of these two classes. Finally, to concretely illustrate the differences between the Dagger

and Plumber classes, we also provide a toy pipeline in the '`doc/examples`' directory of the source-code distribution, with the same directed acyclic graph (*i.e.*, same pipeline) implemented as either a Dagger ('`hello_dagger.py`') instance or a Plumber instance ('`hello_plumber.py`'). An example of a more complex Plumber-instantiated workflow is the above (§2.3 above) MD loop-refinement pipeline.

3.3 PaPy in the context of cloud computing

It should, in principle, be feasible to deploy PaPy workflows in cloud-computing environments without any modifications to the current PaPy codebase. This is because, at the level of PaPy, compute resources are highly abstracted (as NuMap objects) such that a pipeline can be executed using any configuration of resources – local desktops, remote workstations, or a mixture thereof. As described in the text, PaPy employs RPyC to distribute a pipeline across networked computers. Though RPyC is not officially 'advertised' as a tool for cloud computing, it is meant as "*a library for remote procedure calls, clustering and distributed-computing.*" (See the RPyC homepage at http://rpyc.wikidot.com). Indeed, RPyC's basic goal of providing "*remote machines as if they were local resources*" (http://www.ibm.com/developerworks/linux/library/l-rpyc) – *i.e.*, virtualizing resources – can be considered as cloud computing (the terminology is still somewhat fuzzy and non-standardized). In terms of PaPy and its relationship to RPyC, we note that the cloud should be readily accessible by employing a Python-aware service provider such as PiCloud (http://www.picloud.com). The PiCloud service essentially offers a high-level wrapper that uses Amazon Web Services' *Elastic Compute Cloud* (EC2) as the underlying compute resource. PiCloud supplies users with a Python library ('cloud') for seamless integration with another code-base (*e.g.*, a user's PaPy-enabled workflow). Under such a scheme, PaPy pipelines would be rendered cloud-compatible by simply importing the 'cloud' module and using it to dispatch the PaPy workflow, as in the following code snippet:

```
def worker_function(inbox, param):
    # ordinary definition of PaPy worker function...
def picloud_function():
    # makes something using inbox
cloud.call(picloud_function)
```

Similarly, PaPy workflows should also be cloud-compatible with other service providers (*e.g.*, the lower-level EC2 itself), without any necessary modifications to PaPy. A key consideration would be that PaPy assumes data are transmitted across a *stable* internet connection (*e.g.*, on LANs, such as in academic labs or a collection of workstations in a department) without network time-outs, firewall blocks, intermittency problems, *etc*. In this regard, we note that PiCloud assures a "*highly robust computing environment*" with "*99.9% availability*" (see the PiCloud website).

4 A brief, comparative overview of PaPy and KNIME

Although a complete analysis of the similarities and differences between PaPy and the many existing WMS (and WMS-related) software packages lies beyond the scope of this report, we note that currently available WMS programs (free and commercial) have been recently reviewed (see main text); for instance, Tiwari & Sekhar's treatment (5), which emphasizes workflow suites from a biosciences perspective, includes useful summaries of workflow solutions and workflow-compatible third-party software (existing programs that are readily integrated as nodes in different workflow suites). As introduced in the *Background* section of our main text, workflow solutions can be categorized and classified based upon several different criteria – their feature sets and scope of functionality, target application domains, graphical *versus* script-based workflow composition, *etc*. From a user perspective, a most basic distinction is in the intended purpose/scope of the workflow software, with many WMS systems serving as all-inclusive, highly-integrated suites with high-level feature sets ('heavyweight' solutions). In contrast, 'lightweight' solutions (*libraries*, *toolkits*) provide

neither as extensive a set of functionality (at least not out-of-the-box) nor visual environments for workflow composition and enactment, but they do offer the benefits of being *(i)* quite flexible and applicable in a variety of problem domains, *(ii)* more easily mastered by new users, and *(iii)* more transparent in operation, so performance can be more readily optimized and troubleshooting can be more easily pursued. Thus, with scientific workflow solutions it is not the case that "one size fits all" (6), and different approaches – heavyweight suites, lightweight libraries – are more naturally suited to different purposes. Whereas PaPy belongs to the class of lightweight toolkits for workflow/pipeline construction (of which there are not many available packages), KNIME is an example of a comparably heavyweight solution, originally inspired by needs arising in machine learning and data-mining workflows (7). KNIME and PaPy differ in rather fundamental ways – in terms of their intended purposes and scope, their feature sets, and their low-level software implementations.

KNIME, which is written in Java and implemented as an Eclipse plug-in (7), is a feature-rich, all-encompassing workflow/pipelining engine that emphasizes a graphical approach to workflow creation and execution. In the words of its authors (7), KNIME is "*a modular data analysis environment*" that was "*designed as a teaching, research and collaboration platform*". PaPy, in contrast, serves far more modest purposes: Written in Python using functional programming paradigms, PaPy provides a much lower-level, lightweight toolkit. Users can flexibly employ PaPy, together with their own data-analysis and data-processing code (Python or otherwise), in order to distribute calculations locally and/or remotely, improve their data-handling and provenance (have a record of the exact data-processing pipeline), and so on (see main text). Perhaps most important in practical terms, PaPy can be learned by users already familiar with basic programming (at the level of bioinformatic scripting) with relatively minimal effort; starting with the examples we provide, such users can begin creating distributed data-processing pipelines within a day, and can easily adapt their processing pipelines for subsequent projects. Starting with no prior experience with workflow editors and environments, a greater time investment may be required for such users to reach the same level of mastery with a heavyweight, all-encompassing suite such as TAVERNA or KNIME. Thus, considered in terms of the usual trade-offs between functionality/scope/simplicity, both types of systems – lightweight (PaPy) and heavyweight (KNIME, TAVERNA, *etc.*) – exhibit relative strengths and weaknesses, and can be viewed as complementary approaches that serve somewhat different purposes.

Beyond major differences in scope and feature-set, PaPy and heavyweight WMS suites such as KNIME differ at other levels too. In terms of low-level software implementation, PaPy and KNIME employ fundamentally different execution models: Whereas PaPy nodes ('pipers') process continuous streams of data-items (see text), each discrete node in a KNIME workflow processes an entire 'chunk' of data before forwarding the resultant data to successive (downstream) nodes (8). In addition, whereas PaPy treats intermediate data as serialized Python objects (which can be arbitrarily complex, so long as they are valid data structures), KNIME wraps all inter-node data (*i.e.* as they traverse the data-flow) as custom *key*/*value* (table-like) data structures. KNIME and PaPy also differ substantially in their approach to distributing pipelines for execution across a network of computers (see text for PaPy's approach), with much recent effort having been devoted to the development of a KNIME 'Grid Engine' for distributed processing (9). Finally, because both KNIME and PaPy ultimately treat the problem of data-processing using a pipeline approach, they share certain similarities in terms of load-balancing and performance tuning. For instance, the factors influencing the value to which PaPy's '*stride*' parameter is set (namely, the memory/speed-up trade-off) are mirrored in the fact that "*a suitable balance between the size and the number of chunks*" is important in KNIME too (8).

References

[1] Anishkin A, Milac AL, Guy HR: **Symmetry-restrained molecular dynamics simulations improve homology models of potassium channels.** *Proteins* 2010, **78**(4):932–949.

[2] Frishman D, Argos P: **Knowledge-based protein secondary structure assignment.** *Proteins* 1995, **23**(4):566–579.

[3] Hinsen K: **The molecular modeling toolkit: A new approach to molecular simulations**. *Journal of Computational Chemistry* 2000, **21**(2):79–85.

[4] Pieper U, Eswar N, Braberg H, Madhusudhan MS, Davis FP, Stuart AC, Mirkovic N, Rossi A, Marti-Renom MA, Fiser A, Webb B, Greenblatt D, Huang CC, Ferrin TE, Sali A: **MODBASE, a database of annotated comparative protein structure models, and associated resources.** *Nucleic Acids Res* 2004, **32**(Database issue):D217–D222.

[5] Tiwari A, Sekhar AKT: **Workflow based framework for life science informatics.** *Comput Biol Chem* 2007, **31**(5-6):305–319.

[6] Curcin V, Ghanem M: **Scientific workflow systems - can one size fit all?** In *Proc. Cairo Int. Biomedical Engineering Conf. CIBEC 2008* 2008:1–9.

[7] Berthold MR, Cebron N, Dill F, Fatta GD, Gabriel TR, Georg F, Meinl T, Ohl P, Sieb C, Wiswedel B: **KNIME: the Konstanz Information Miner**. In *Proceedings 4th Annual Industrial Simulation Conference, Workshop on Multi-Agent Systems and Simulation (ISC 2006)*, Palermo, Italy 2006.

[8] Sieb C, Meinl T, Berthold MR: **Parallel and Distributed Data Pipelining with KNIME**. *The Mediterranean Journal of Computers and Networks* 2007, **3**(2):43–51.

[9] Berthold MR, Cebron N, Dill F, Gabriel TR, Kötter T, Meinl T, Ohl P, Thiel K, Wiswedel B: **KNIME - The Konstanz Information Miner**. *SIGKDD Explorations* 2009, **11**.
